# Levosimendan: use, cost-effectiveness and outcome in a tertiary cardiothoracic centre

**DOI:** 10.1186/cc14232

**Published:** 2015-03-16

**Authors:** A Ranjan, N Bhudia, I McGovern, C Walker, L Kuppurao

**Affiliations:** 1 Royal Brompton & Harefield NHS Foundation Trust, Harefield, London, UK

## Introduction

Levosimendan was originally developed for the treat ment of decompensated heart failure in situations for which conventional therapy is not sufficient. It is an effective calciumsensitising drug with vasodilatory and inotropic effects and improves cardiac contractility. Trials have shown positive outcome benefit with the use of levosimendan [1]. We reviewed the usage levosimendan at our institution and outcome of these patients.

## Methods

**Figure 1:**
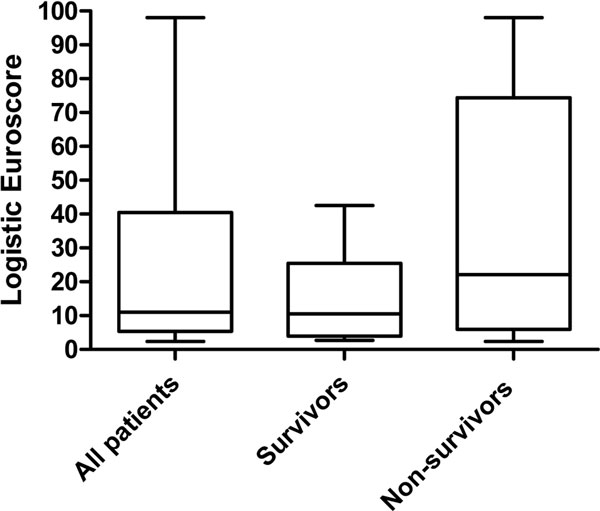
**Logistic EuroSCORE of all patients**.

We reviewed the use of levosimendan at Harefield from January 2013 through December 2013. Patient demographics, logistic EuroSCORE (Figure 1), diagnosis, surgical or intervention details, inotropic support, dosage and duration of levosimendan use, length of stay in the ICU, cost (Table 1) and patient outcome were collected.

**Table 1:** Cost of levosimendan based on a 70 kg patient.

	Levosimendan	Adrenaline	Milrinone
Maximum dose (μg/kg/minute)	0.2	1	0.7
Cost per vial (£)	894	2.30	16
Cost per 24 hours (£)	894	7	112

## Results

Levosimendan was used in 30 patients, 23 (77%) male and seven (23%) female. Median age was 69 (59 to 72.8). Levosimendan was used post cardiac surgery, post angioplasty and patients with ventricular assist devices (VAD) and extracorporeal membrane oxygenator (ECMO). Most of the patients received a standard regimen of 12.5 mg administered at a dose of 0.1 μg/kg/minute for 24 hours. Concurrent noradrenaline was used in most of the patients ranging from 0.02 to 0.2 μg/kg/minute. The median length of stay in the ICU was nine (6 to 14.5) days for survivors and 23.5 (7.5 to 36) days in nonsurvivors. Sixteen patients (55%) survived and were discharged from the hospital.

## Conclusion

We have successfully used this drug in high-risk patients during the perioperative period with good results without major complications. Levosimendan seems to reduce catecholamine requirement, the need for mechanical circulatory support, and the duration of critical care, which can justify the cost of this drug. It can be also useful in weaning patients from short-term VAD and ECMO. Larger studies are required in this area.
